# Joint Association of Education and Neighborhood Socioeconomic Status with Smoking Behavior: The Multiethnic Cohort Study

**DOI:** 10.21203/rs.3.rs-5281444/v1

**Published:** 2024-11-01

**Authors:** Catherine P. Walsh, Salma Shariff-Marco, Younghan Lee, Lynne R. Wilkens, Loic Le Marchand, Christopher A. Haiman, Iona Cheng, S. Lani Park

**Affiliations:** University of Hawaii Cancer Center; University of California, San Francisco; University of Hawaii at Mānoa; University of Hawaii Cancer Center; University of Hawaii Cancer Center; University of Southern California; University of Southern California; University of Hawaii Cancer Center

**Keywords:** Smoking, neighborhood, SES, education, prevalence, multiethnic

## Abstract

**Background:**

Cigarette smoking is the leading cause of preventable mortality. Both neighborhood- and individual-level socioeconomic status (SES) are inversely associated with smoking. However, their joint effect on smoking behavior has not been evaluated.

**Methods:**

This cross-sectional study examined the association of education and neighborhood SES (nSES) with smoking among 166,475 Multiethnic Cohort (MEC) participants (African American, Japanese American, Latino, Native Hawaiian, White individuals) recruited between 1993–1996 from Hawaii and LA County. nSES was based on a composite score of 1990 US Census data and assigned to geocoded addresses; nSES quintiles were based on region-specific distributions. The joint education/nSES variable had four categories: high nSES (Quintiles 4–5)/high education (> high school), high nSES/low education (≤ high school), low nSES (Quintiles 1– 3)/high education, and low nSES/low education. Poisson regression estimated state-specific prevalence ratios (PR) for current smoking versus non-smoking across joint SES categories, with subgroup analyses by sex and race/ethnicity.

**Results:**

In California, compared to MEC participants with high nSES/high education, the PR for smoking was highest for low nSES/low education (PR = 1.50), followed by low nSES/high education (PR = 1.33) and high nSES/low education (PR = 1.29). All pairwise comparisons between PR were statistically different (*p* < 0.0001), except high nSES/low education vs. low nSES/high education. In Hawaii, compared to high nSES/high education, the PR for smoking was also highest for low nSES/low education (PR = 1.41), but followed by high nSES/low education (PR = 1.36), then low nSES/high education (PR = 1.20). All pairwise comparisons were statistically different (*p* < 0.0001), except high nSES/low education vs. low nSES/low education. These patterns were consistent across sex and race/ethnicity within each state.

**Conclusion:**

In California and Hawaii, individuals with low education living in low SES neighborhoods had the highest smoking prevalence. However, regional differences were noted: in California, both low education and low nSES increased smoking prevalence; whereas in Hawaii, low education had a greater impact.

## 1. Introduction

Cigarette smoking is the leading cause of preventable mortality in the United States, accounting for approximately 1 in 5 deaths every year^1^. Smoking is a primary risk factor for many common causes of death including heart disease, multiple cancers, and chronic obstructive pulmonary disease^2^. Despite declines in smoking prevalence across the United States (US) from 42% in 1965 to 12.5% in 2020, nearly 30.8 million adults in the US smoked cigarettes in 2020^3,4^. Notably, smoking prevalence differs across populations. For instance, current smoking prevalence is higher in Southern states compared to Western states, among males compared to females, and among individuals with a high school diploma or less compared to those with more education than a high school diploma, and other measures of socioeconomic status^1,3,5,6^. Current smoking prevalence also differs across race and ethnicity. Indeed, current smoking in the US is disproportionately high among racial and ethnic minoritized groups, including African American, American Indian/Alaska Native, Native Hawaiian and Pacific Islander, and non-Hispanic non-White individuals who self-identify with 1 or more racial or ethnic minoritized groups^3,4,6–8^. Further, among those who currently smoke cigarettes, American Indian/Alaska Native, Native Hawaiian, and non-Hispanic White individuals smoke more cigarettes per day compared to Hispanic and Asian American individuals^9,10^. The reasons for these racial and ethnic disparities in smoking rates and intensity cannot be explained by individual level factors alone without recognizing the influence of upstream factors, including the neighborhood contextual environment^11^.

Prior studies have shown that individual-level socioeconomic status (SES) measures, such as educational attainment, occupation, or income do not fully explain racial and ethnic disparities in smoking prevalence or intensity^12–16^. Thus, recent attention has turned to upstream contextual SES factors, such as neighborhood socioeconomic status (nSES), as a social driver of health that may influence racial and ethnic disparities in smoking behavior. Measures of nSES that are a composite of census-based data include multiple socioeconomic domains (e.g., income, employment/occupation, education, housing) in a specified geographic area^17,18^, provide a more comprehensive assessment of nSES and/or area disadvantage. Prior research using such measures has shown that residing in lower versus higher SES neighborhoods is associated with higher prevalence of current smoking status, even after adjustment for individual-level SES indicators such as education^12,14,19–21^. For example, in a study of primarily White individuals from Maryland, North Carolina, and Minnesota (n = 9,580), and Black individuals from Mississippi (n = 3,021), greater neighborhood disadvantage compared to less neighborhood disadvantage was associated with higher prevalence of current smoking for both White and Black individuals, and this difference persisted after adjustment for individual educational attainment^14^. Other studies suggest these associations may differ across racial and ethnic groups and geography. For example, in the Coronary Artery Risk Development in Young Adults (CARDIA) study, comprised of roughly equal numbers of Black and White individuals (n ≈ 1,700 in each group, age 28–40 years) from the Southern US, Midwest, and California, White individuals living in the least advantaged compared to the most advantaged areas had a higher smoking prevalence, after adjustment for individual-level income, education, and occupation^21^. However, for Black individuals living in the least advantaged areas, smoking prevalence was high only among a subset with higher levels of individual income^21^, pointing to the importance of assessing the joint association of income and nSES on smoking behavior. Together, these findings underscore the need to explore how individual-level and neighborhood-level indicators of SES interact in association with smoking behavior. Indeed, current conceptual frameworks propose that health disparities arise from the interplay between the broader social environment and individual risk factors^9^. Here, prior work using a joint measure of education and nSES has been effective in revealing the combined effect of nSES, education, and race and ethnicity on health outcomes such as all-cause and cause-specific morbidity and mortality^20–22^.

Thus, although there is a growing body of literature suggesting that both neighborhood and individual-level SES factors may jointly influence smoking behavior^13,23–26^, with potential differences by race and ethnicity, there is limited knowledge about these relationships in large population-based samples that include disaggregated Asian American and Native Hawaiian groups. Here, we examine the joint association of nSES and education with smoking status in over 160,000 participants from the Multiethnic Cohort (MEC).

## 2. Methods

### 2.1 Study population

The MEC is a prospective cohort study that was initiated in 1993–1996 and has been described in detail previously^29^. The cohort of >215,000 participants is primarily comprised of individuals who self-identified as African American, Japanese American, Latino, Native Hawaiian, and White. At enrollment, participants were 45 through 75 years and were recruited from California, primarily Los Angeles County, and the state of Hawaii. Participants were initially identified in California and Hawaii through driver’s license files, as well as Health Care Financing Administration files in California and voter registration lists in Hawaii. Eligible participants completed a mailed, self-administered questionnaire assessing information regarding demographic, anthropometric, medical history, family history, reproductive history, diet, and lifestyle factors (e.g., smoking, physical activity). The Institutional Review Boards at the University of Southern California and University of Hawaii approved the study protocol.

From the >215,000 participants enrolled in MEC, we excluded individuals who were not among one of the five main race and ethnicity groups (n = 13,987), had no follow-up time (n = 7), or had a prior history of lung cancer (identified by self-report or linkage to cancer registries in California or Hawaii; n = 725). Participants were also excluded if their addresses could not be geocoded (n = 7,842), or if they were missing nSES, education, or smoking status data (n = 6,335). After removing those with missing data for any included covariates (n = 20,278), there were 166,475 participants remaining for analyses.

### 2.2 nSES and education exposures

Geocoding of participant addresses at baseline (1993–1996) and the development of the nSES index has been described previously^17,26,30^. In brief, we used an established measure of nSES that is a composite index derived from principal component analysis of U.S. census block group data (1990) on education, occupation, employment, household income, poverty, and rental and house values for LA County (California) and Hawaii^17,18^. We categorized nSES into quintiles based on regional distributions (state for HI and LA county for CA), and all analyses were conducted separately by region^26^. Low and high nSES were defined as quintiles 1 to 3 and 4 to 5, respectively.

Educational attainment was self-reported on the baseline questionnaire^29^ and was categorized as: high school graduate or less, vocational school or some college, college graduate, or graduate and professional school. Low and high education were defined as high school graduate or less (≤ 12 years of education) and greater than high school graduate (> 12 years education), respectively. A joint neighborhood and education SES measure was created with four categories: high nSES and high education; high nSES and low education; low nSES and high education; and low nSES and low education.

### 2.3 Smoking Outcomes

Smoking status was self-reported on the baseline questionnaire^29^ and categorized as individuals who smoke (≥ 20 packs of cigarettes in one’s lifetime and still smoke), formerly smoked (≥ 20 packs of cigarettes in one’s lifetime, but quit), or never smoked (< 20 packs of cigarettes in one’s lifetime). A non-smoking category was created by combining individuals who formerly smoked or never smoked. Smoking intensity was assessed among individuals who reported smoking at baseline as the average number of cigarettes smoked per day (CPD; ≤ 5 per day, 6–10 per day, 11–20 per day, 21–30 per day, or ≥ 31 per day). CPD was treated as a continuous variable after categories were assigned the following respective values: 5, 8, 15.5, 25.5, and 31.

### 2.4 Statistical analysis

**Figure 1:**
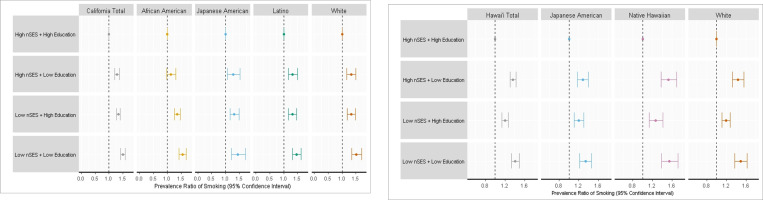
A. Joint association of education and nSES with smoking prevalence at baseline, by race and ethnicity in California, MEC (1993–1996). Models were adjusted for age at cohort entry, sex, race and ethnicity (Total model), marital status, physical activity levels, occupational exposure (industry field (yes/no) and laborer or office professional (yes/no)), alcohol intake (g/day), body mass index (BMI, weight in kg/height in meters squared (kg/m^2^), Healthy Eating Index diet quality (HEI-2015; scored 0–100), and census block group. B. Joint association of education and nSES with smoking prevalence at baseline, by race and ethnicity in Hawaii, MEC (1993–1996). Models were adjusted for age at cohort entry, sex, race and ethnicity (Total model), marital status, physical activity levels, occupational exposure (industry field (yes/no) and laborer or office professional (yes/no)), alcohol intake (g/day), body mass index (BMI, weight in kg/height in meters squared (kg/m^2^)), Healthy Eating Index diet quality (HEI-2015; scored 0–100), and census block group.

We used Poisson regression to estimate the prevalence ratios (PR)^31^ and 95% confidence intervals (CIs) for the associations of smoking status (smoking versus non-smoking) with the joint distribution of education and nSES by study area (California or Hawaii). The minimally adjusted models included age at cohort entry, sex (male, female), and race and ethnicity (African American, Japanese American, Latino, Native Hawaiian, White), and census block group to account for clustering. Fully adjusted models included age at cohort entry, sex (male, female), race and ethnicity (African American, Japanese American, Latino, Native Hawaiian, White), marital status (married, single, separated, divorced or widowed, or unknown)^32^, physical activity levels (in metabolic equivalents of a task (METS) for activities in a typical 24 hour day, relative to 1 for sitting)^33^, work status (with six categories that combine industries and occupations employed for 10 years or more [yes: manufacturing enterprises (i.e., government regulation of manufacturing), or no: none of those enterprises] and longest worked occupation classifications [office work only, labor/craft only, or both])^34^, alcohol intake (grams/day)^33^, body mass index (BMI, weight in kg/height in meters squared (kg/m^2^)^32^), and Healthy Eating Index diet quality (HEI-2015; scored 0–100^35,36^)^33^. Sensitivity analyses were conducted that compared whether associations with SES would change if the outcome was comparing never (Table S1A) or former (Table S1B) smokers to current smokers. Smoking associations with the joint SES variable were similar for both outcome comparisons. Thus, the comparison of those who currently smoke versus non-smoking individuals are the primary findings presented (Figure 1A, 1B; Table 2). Given that the main independent variable (joint SES) was categorical with four levels, we conducted an omnibus Wald test for significance (df = 3), with post-hoc pairwise comparisons using contrast coding for specific comparisons of interest and adjusting for multiple comparisons by Bonferroni correction^37^, where there were six independent tests (*p*=0.05/6 = 0.008). Subgroup analyses were performed by sex and race and ethnicity. Heterogeneity between subgroups was tested using the Wald statistic for the cross-product term of the joint SES measure and subgroup indicator. To compare with prior literature, the independent association of education and nSES with smoking prevalence was modeled using dichotomous variables in main effects models.

We used linear mixed models to assess the associations of smoking intensity (cigarettes per day; CPD) with the joint SES measure among individuals who reported smoking at baseline^38^. Models for smoking intensity included the same covariate adjustment variables, subgroup analyses, and assessment of main effects and interaction as described above.

All analyses were performed using SAS (version 9.4).

## 3. Results

The baseline characteristics of the MEC participants are presented by study area in Tables 1A and 1B^29^. In California (n = 85,092), the population was comprised of Latino participants (40.5%), followed by African American (31.0%), White (14.9%), Japanese American (13.4%), and Native Hawaiian participants (0.2%). In Hawaii (n = 81,383), most of the participants were Japanese American (47.7%), followed by White (37.4%), and Native Hawaiian participants (15.0%). The average age of participants in California and Hawaii was 60.4 and 58.8 years, respectively, with Native Hawaiian participants being the youngest (56.8 and 56.0 years, respectively) and Japanese American participants, the oldest (58.8 and 60.4 years, respectively).

In California, 16.8% of MEC participants self-reported currently smoking at baseline (Table 1A), compared to 15.2% in Hawaii (Table 1B). Among the California MEC participants, smoking prevalence was highest for African American participants (22.9%), followed by Native Hawaiian (20.5%), White (16.8%), Latino (13.9%) and Japanese American participants (11.4%). Among MEC participants in Hawaii, the prevalence of currently smoking was highest in Native Hawaiian participants (22.5%), followed by White (16.3%) and Japanese American participants (12.0%). In both California and Hawaii, among those who smoke, White participants self-reported the highest intensity of smoking compared to participants of other race and ethnicity groups (California: 17.8 ± 8.2 CPD, Table 1A; Hawaii: 19.3 ± 8.1 CPD, Table 1B).

In California, 38.3% of MEC participants were in the low nSES/low education category, followed by 29.3% in the low nSES/high education category, 10.0% the high nSES/low education category, and 22.4% in the high nSES/high education category (Table S2A). In Hawaii, 17.8% of MEC participants were in the low nSES/low education category, followed by 24.3% in the low nSES/high education category, 17.6% in the high nSES/low education category, and 40.2% in the high nSES/high education category and (Table S2B).

### 3.1 Smoking Prevalence ratios

For California, there were significant independent main effects of low nSES (PR = 1.26; 95% CI: 1.21–1.32) and low education (PR = 1.16; 95% CI: 1.12–1.20) on smoking prevalence compared to high nSES and high education, respectively (Table S5A). When considering the joint effect of nSES and education on smoking among California MEC participants, compared to those in the high nSES/high education category, the PR for smoking was highest in the low nSES/low education category (PR = 1.50; 95% CI: 1.42–1.58), followed by low nSES/high education (PR = 1.33; 95% CI: 1.27–1.40) and high nSES/low education (PR = 1.29; 95% CI: 1.21–1.37) (Fig. 1A, corresponding data: Table 2). There was also significant difference across joint SES categories (omnibus p < 0.0001; Table 2). Notably, no statistically significant difference was detected in PRs between the low nSES/high education and the high nSES/low education categories (*p* = 0.24). All other pairwise comparisons were significant (*p*’s < 0.05). A formal test for interaction was significant (*p* < 0.0007; Table 2), indicating that, in California, smoking prevalence was higher when individuals experienced low education and low nSES together compared to the linear combination of these traits (i.e., both independently) from the main effects model.

While there was significant heterogeneity across sex (*p*-het < 0.001; Table S3), in subgroup analyses, the overall pattern of smoking PRs was similar between males and females in California. Across racial and ethnic groups, there was no significant heterogeneity in PRs (*p*-het = 0.08; Table S4A). When we examined the pattern of results *within* racial and ethnic groups in California, there was a significant difference across joint SES categories for each racial and ethnic group (*p’s* < 0.0001; Table S4A). For Japanese American, Latino, and White individuals, the pattern of PRs was similar to the overall pattern of results (Table S4A). In divergence from the overall pattern, for African American individuals, there was a significant difference between PRs for the low nSES/high education and high nSES/low education categories (*p* = 0.01), and the PRs were similar for the high nSES/high education and the high nSES/low education categories (*p* = 0.11).

For Hawaii, there was a significant main effect of low nSES (PR = 1.13; 95% CI: 1.08–1.17) and low education (PR = 1.27; 95% CI: 1.22–1.31) on smoking prevalence (Table S5A). When considering the joint effect of nSES and education on smoking prevalence, compared to Hawaii MEC participants in the high nSES/high education category, the PR for smoking was highest among individuals in the low nSES/low education category (PR = 1.41; 95% CI: 1.33–1.49), followed by high nSES/low education (PR = 1.36; 95% CI: 1.30–1.43) and low nSES/high education (PR = 1.20, 95% CI:1.14–1.27) (Fig. 1B, corresponding data: Table 2). These findings differed from those in California in that the highest PRs were among individuals in both joint SES categories that included low education. Specifically, across joint SES categories, there was a significant difference (omnibus *p* < 0.0001; Table 2), and among those with low education, low nSES areas compared to high nSES areas did not further increase smoking prevalence (*p*_low nSES/low education vs. high nSES/low education_ = 0.20). However, among individuals with high education, low nSES increased smoking prevalence (*p*_low nSES/high education vs high nSES/high education_ < 0.0001). A formal test for interaction was significant (*p* < .0001; Table 2), indicating that, for smoking in Hawaii, smoking prevalence was higher when individuals experienced low education and low nSES together compared to the linear combination of these traits (i.e., both independently).

There was no heterogeneity across sex (*p*-het = 0.101; Table S3) in Hawaii. Across racial and ethnic groups, there was significant heterogeneity (*p*-het < 0.001; Table S4B), however, in subgroup analyses, the overall pattern of PRs was similar for each racial and ethnic group (Fig. 1B, corresponding data: Table S4B). When we examined the pattern of results within racial and ethnic groups in Hawaii, for Japanese American individuals, the PRs for the high nSES/low education category and the low nSES/high education category were additionally similar (*p* = 0.11).

### 3.2 Smoking Intensity

Among those who self-reported smoking at MEC baseline in California, significant independent main effects were observed on smoking intensity for low nSES (β (SE) = −0.50 (0.15), *p* = 0.001) and low education (β (SE) = −0.38 (0.14), *p* = 0.006), compared to high nSES and high education, respectively (Table S10). Compared to individuals in the high nSES/high education category in California (CPD; Mean_adj_ (SE) = 14.3 (0.3)), individuals in the low nSES/low education category smoked the fewest of cigarettes per day (CPD; Mean_adj_ (SE) = 13.4 (0.3), *p* < 0.001), followed by low nSES/high education (Mean_adj_ (SE) = 13.9 (0.3), *p* = 0.03), and then high nSES/low education (Mean_adj_ (SE) = 14.1 (0.3), *p* = 0.42) (omnibus *p* = 0.0001; Table S5). A formal test for interaction was not significant (*p* = 0.44; Table S6).

Among those in California, heterogeneity across sex was significant (*p*-het = 0.02; Table S7), and was suggestive of males smoking more CPD than females; however, the overall pattern of association for nSES and education with CPD was similar between males and females in California. We also observed significant heterogeneity of effects across race and ethnicity in California (*p*-het = 0.01; Table S8). Specifically, the association with CPD smoked among individuals with low nSES/low education compared to those with high nSES/high education was primarily observed among African American (*p* = 0.06) and Latino individuals (*p* < .0001) (Table S8).

In Hawaii, there were no significant independent main effects of low nSES (β (SE) = 0.04 (0.15), *p* = .0.82) or low education (β (SE) = 0.09 (0.15), *p* = 0.55) on smoking intensity (Table S10) and no significant associations with smoking across levels of the joint SES exposure variable were detected (omnibus *p*-value = 0.78; Table S6).

## 4. Discussion

This cross-sectional study examined the association of a joint SES measure, comprised of nSES and individual-level educational attainment, with smoking behavior among a racially and ethnically diverse population living in California, primarily LA County, and Hawaii. In California, compared to individuals with greater than a high school degree (i.e., “high education”) and living in a low SES neighborhood, individuals with a high school education or less (i.e., “low education”) and living in a low SES neighborhood had the highest smoking prevalence, followed by individuals with high education living in a low SES neighborhood, and individuals with low education living in a high SES neighborhood. These patterns of association were largely consistent across sex and racial and ethnic groups in California. In Hawaii, smoking prevalence was highest among individuals with low education, whether or not they lived in a low or a high SES neighborhood. In addition, among individuals with high education, living in a low SES area was associated with a higher smoking prevalence compared to those living in a high nSES area. These patterns of association in Hawaii were consistent across sex and all racial and ethnic groups.

In both California and Hawaii, individuals with low education who were living in low nSES areas had the highest smoking prevalence across sex and all racial and ethnic groups. Specifically, individuals with low education living in low nSES areas had a 50% higher prevalence of smoking in California and a 41% higher prevalence of smoking in Hawaii compared to individuals with high education living in high nSES areas. While these estimates are not directly comparable to prior studies, our estimates of smoking prevalence for nSES and education independent of one another are commensurate. In California, smoking prevalence was 26% higher among those in low compared to high nSES areas, and 16% higher among individuals with low compared to high education. In Hawaii, these figures were 13% and 27%, respectively. These estimates are consistent with prior studies in the US that investigated smoking prevalence with either individual-level or neighborhood-level measures of SES^12–15,19^. For example, in the Southern Community Cohort Study (SCCS), which is comprised of > 70,000 African American and White individuals living in the Southern US, investigators found that within each race and sex group, those with the lowest education (< 9 years), compared to the highest education (≥ 16 years), had a higher prevalence of smoking (PR’s = 1.14–1.62), after controlling for other individual-level SES indicators such as income^13^, but not neighborhood factors. The SCCS also reported that, within each race and sex group, there was a higher prevalence of smoking (PR’s = 1.05–1.24) among those with the lowest neighborhood advantage score (computed using area-based measures of income, housing, education, and occupation) compared to the highest advantage score, after adjustment for individual-level SES indicators^13^. Thus, while the overall pattern of findings in our study align with prior studies examining either individual-level or neighborhood-level SES measures in relation with smoking prevalence, we provide unique estimates for the joint education and neighborhood-level SES association with smoking prevalence across two geographic regions (Hawaii and LA County, CA) that contain high racial and ethnic diversity.

Among African American individuals in California, living in a low SES neighborhood had a greater influence on current smoking status compared to low educational attainment. Specifically, smoking prevalence was 36% higher for those living in low compared to high nSES areas, among those with high education. These estimates are consistent with those reported in the SCCS, with a 5–24% higher smoking prevalence among Black individuals with the lowest compared to the highest neighborhood advantage score, after accounting for individual level factors^13^. Notably, in California, smoking prevalence among African American individuals with low education was not significantly higher than smoking prevalence of those with high education, living in high nSES areas. This is in contrast with findings reported in the SCCS, with a statistically significant 14–27% higher smoking prevalence for Black individuals with the lowest compared to the highest level of education (< 9 years vs. ≥ 16 years)^11^. This difference may reflect differences in measurement between the two studies, but could also reflect differences in the lived experiences of African American individuals residing in California compared to the Southern US in structural and social drivers of health, such as historical and contemporary redlining, unequal access to quality education and resources, and targeted tobacco marketing in African American neighborhoods^39–42^. However, given that ours is the first report of this finding, our results should be interpreted with caution.

Across all racial and ethnic groups in Hawaii, we found that educational attainment had a strong influence on smoking prevalence, although low nSES also played a role. Specifically, for all participants in Hawaii, we found a 36% increase in smoking prevalence associated with low education compared to high education, among individuals living in high nSES areas. In addition, the joint exposure of low education and low nSES together did not significantly increase the smoking prevalence associated with low education among those in high nSES areas. Prior research in Hawaii, with population samples that include East Asian, Native Hawaiian, and White middle aged adults (41–73 years of age), have also reported that lower education levels (i.e., high school level or less) were associated with greater smoking prevalence^32,43^. While these studies acknowledge that Native Hawaiian individuals experience greater poverty and unemployment in Hawaii compared to East Asians and White individuals, which likely contributes to greater smoking prevalence^4,32^, these studies did not examine nSES. In our study, we found a 20% higher smoking prevalence associated with living in low nSES compared to high nSES areas, among individuals with high education. To our knowledge, ours is the first study to investigate and report on the impact of nSES on smoking behavior in Hawaii. Thus, our findings suggest that obtaining higher levels of education, beyond high school, may be important in reducing cigarette smoking rates in Hawaii, while also highlighting the need for further research into neighborhood-level factors that may contribute to smoking behavior, such as structural racism, poverty, and exposure to tobacco marketing.

In an examination of daily smoking intensity, we found that MEC participants living in low nSES areas in California with a low education smoked fewer cigarettes per day (CPD), on average, compared to individuals living in high nSES areas with high education. Using data from the US National Health Interview Survey (NHIS), individuals of the same age range as those in the MEC at the time of study (i.e., 40–65 years in ~ 1990) similarly reported lower intensity smoking with lower levels of education (i.e., less than high school)^44^. However, not all findings are consistent. For example, using data from the Current Population Survey Tobacco Use Supplement (n = 19,004), consisting of primarily White and Hispanic individuals ages 18–64 years (with 36.2% ages 45–64 in 1992–1993), overall daily cigarette consumption was lower in California compared to the rest of the US; however, for both the broader US and California specific samples, individuals with less than a high school education had a three times greater odds of being a heavy smoker (≥ 20 cigarettes /day; OR = 3.28) compared to college graduates^45^. Interestingly, neither of these studies accounted for area-level measures of SES. When considering the role of neighborhood context, the Multi-Ethnic Study of Atherosclerosis (MESA), a population-based sample comprised of 6,814 adults aged 44–84 years drawn from six US cities across California, the Midwest, and the East Coast, found no association between a neighborhood-level SES variable (i.e., social environment score) and smoking intensity, after controlling for education^46^. When our analyses were stratified by race and ethnicity, the overall pattern of association was particularly strong among African American and Latino individuals. Other studies among individuals of the same age range similarly report that Black and Hispanic/Latino individuals who smoke are more likely to smoke with less intensity (fewer CPD) compared to non-Hispanic White individuals^45,47–49^, after accounting for education. Thus, our study adds to the literature regarding lower smoking intensity among Black and Latino individuals with low education, but is unique in demonstrating that African American and Latino individuals living in a low SES neighborhood in California with low education smoked fewer CPD.

A major strength of this study was the ability to account for a number of potential confounders in the relationship between neighborhood- and individual-level SES and smoking prevalence, including alcohol intake and other lifestyle factors^32,33^. Our population sample is also unique in that it includes a large racially and ethnically diverse population across two different geographic regions in the US: LA County, California and Hawaii. Our analytic sample also had a large number of individuals in each category of nSES and educational attainment, both within and between racial and ethnic groups. This allowed examination of differences across these factors using a joint variable of nSES and education with both smoking prevalence (N = 166,475) and intensity (CPD; n = 26,656). However, our study was not without limitations. First, the analyses here are cross-sectional and temporality cannot be assessed. Second, our analyses do not consider the use of other nicotine products, such cigars or smokeless tobacco^50^, which could influence overall cigarette use among different racial and ethnic groups^51^. However, the prevalence of smokeless tobacco use in the US population is much lower than cigarette use^50^. Third, although we used the same indicators for nSES in California and Hawaii, there was variation in the distribution of individual nSES components between states; thus, while similar, nSES in our study is not absolutely comparable between states. Fourth, census block groups are based on administrative boundaries and may not reflect how study participants define their neighborhoods. However, census tracts and block groups have been indicated as reasonable proxies for neighborhoods when conducting population-based studies such as this one^52^. Finally, our data were collected in the mid-1990’s and the determinants of currently smoking in adults may be somewhat different now.

In conclusion, the use of a joint SES measure consisting of educational attainment and neighborhood-SES suggests that the joint exposure to low education and low nSES together had the greatest influence on smoking prevalence in this multiethnic population from California and Hawaii. Differences were noted across the two geographic regions. Specifically, in California, both low education and low nSES independently increased smoking prevalence, while in Hawaii, exposure to low education had the greatest influence on smoking prevalence. Notably, low nSES increased smoking prevalence among those with high education in Hawaii, suggesting that nSES may play an independent role in promoting smoking among individuals with high levels of education in Hawaii. Future research should continue to explore the coexisting and intersecting inter-relationships among nSES, education, and race and ethnicity in association with smoking behavior, including replicating our findings and extending to populations that include other US regions and age ranges.

## Supplementary Material

Supplement 1

## Figures and Tables

**Table 1A.** Characteristics of participants in California, by race and ethnicity, MEC (1993–1996)

	California											
	Race and Ethnicity, N (%)											
	African American		Japanese American		Latino		Native Hawaiian		White		Total	
**Characteristic**	26350	(31.0)	11421	(13.4)	34490	(40.5)	156	(0.2)	12675	(14.9)	85092	(100.0)
Age (years)												
mean (sd)	60.5	(9.0)	61.7	(8.8)	59.6	(7.7)	56.8	(7.4)	61.2	(8.2)	60.4	(8.4)
Sex, N (%)												
male	9736	(36.9)	5628	(49.3)	17348	(50.3)	85	(54.5)	4552	(35.9)	37349	(43.9)
female	16614	(63.1)	5793	(50.7)	17142	(49.7)	71	(45.5)	8123	(64.1)	47743	(56.1)
Marital Status, N (%)												
married	12430	(47.2)	8590	(75.2)	23734	(68.8)	113	(72.4)	8207	(64.7)	53074	(62.4)
single	1830	(6.9)	999	(8.7)	2116	(6.1)	11	(7.1)	935	(7.4)	5891	(6.9)
separated, divorced, or widowed	12090	(45.9)	1832	(16.0)	8640	(25.1)	32	(20.5)	3533	(27.9)	26127	(30.7)
BMI^a^ (kg/m^2^)												
mean (sd)	28.5	(5.8)	24.0	(3.5)	27.8	(4.8)	27.0	(5.0)	26.7	(5.3)	27.3	(10.9)
Diet Quality (HEI-2015 units^b^)												
mean (sd)	69.5	(10.6)	64.8	(9.8)	65.3	(9.6)	63.2	(9.8)	68.5	(10.7)	67.0	(10.3)
Physical Activity (METS/day^c^)												
mean (sd)	1.6	(0.3)	1.6	(0.3)	1.7	(0.3)	1.6	(0.4)	1.6	(0.3)	1.6	(0.3)
Alcohol intake (g/day^d^)												
mean (sd)	8.1	(26.4)	5.4	(16.7)	8.5	(26.7)	10.0	(29.6)	9.9	(24.0)	8.2	(25.1)
Smoking Status^e^, N (%)												
Non-smoking	20303	(77.0)	10118	(88.6)	29693	(86.1)	124	(79.5)	10542	(83.2)	70780	(83.2)
never smoked	9832	(37.3)	5464	(47.8)	16698	(48.4)	68	(43.6)	5222	(41.2)	37284	(43.8)
formerly smoked	10471	(39.7)	4654	(40.8)	12995	(37.7)	56	(35.9)	5320	(42.0)	33496	(39.4)
Current Smoking	6047	(23.0)	1303	(11.4)	4797	(13.9)	32	(20.5)	2133	(16.8)	14312	(16.8)
Cigarettes per day, among individuals who reported they currently smoke												
mean (sd)	12.9	(6.8)	14.9	(7.3)	11.2	(6.8)	12.8	(6.2)	17.8	(8.2)	13.3	(7.4)
Work Status (employment in a manufacturing enterprise and occupational category), N (%)												
No and office	13544	(51.4)	7563	(66.2)	10857	(31.5)	79	(50.6)	8017	(63.3)	40060	(47.1)
No and labor/craft	3012	(11.4)	734	(6.4)	5976	(17.3)	20	(12.8)	801	(6.3)	10543	(12.4)
No and office/labor/craft	6124	(23.2)	1613	(14.1)	9203	(26.7)	31	(19.9)	2391	(18.9)	19362	(22.8)
Yes and office	954	(3.6)	696	(6.1)	1537	(4.5)	14	(9.0)	677	(5.3)	3878	(4.6)
Yes and labor/craft	2116	(8.0)	618	(5.4)	5543	(16.1)	10	(6.4)	611	(4.8)	8898	(10.5)
Yes and office/labor/craft	600	(2.3)	197	(1.7)	1374	(4.0)	2	(1.3)	178	(1.4)	2351	(2.8)
Education, N (%)												
Low Education (≤ high school)	10060	(38.2)	3388	(29.7)	23109	(67.0)	61	(39.1)	4486	(35.4)	41104	(48.3)
High Education (> high school)	16290	(61.8)	8033	(70.3)	11381	(33.0)	95	(60.9)	8189	(64.6)	43988	(51.7)
nSES Quintile^f^, N (%)												
Low nSES (Quintiles 1–3)	20842	(79.1)	4628	(40.5)	26377	(76.5)	84	(53.8)	5540	(43.7)	57471	(67.5)
High nSES (Quintiles 4–5)	5508	(20.9)	6793	(59.5)	8113	(23.5)	72	(46.2)	7135	(56.3)	27621	(32.5)
Joint SES Exposure^g^, N (%)												
1. Low nSES / Low Education	9011	(34.2)	1796	(15.7)	19163	(55.6)	38	(24.4)	2554	(20.1)	32562	(38.3)
2. Low nSES / High Education	11831	(44.9)	2832	(24.8)	7214	(20.9)	46	(29.5)	2986	(23.6)	24909	(29.3)
3. High nSES / Low Education	1049	(4.0)	1592	(13.9)	3946	(11.4)	23	(14.7)	1932	(15.2)	8542	(10.0)
4. High nSES / High Education	4459	(16.9)	5201	(45.5)	4167	(12.1)	49	(31.4)	5203	(41.0)	19079	(22.4)

Notes:

a BMI: Body Mass Index, derived from self-reported weight in kg divided in self-reported height in meters squared;

b units = points on the healthy eating index-2015: 0–100; Krebs-Smith et al., 2018; Park et al., 2021;

c metabolic equivalents of a task (METS) for activities in a typical 24 hour day, relative to 1 for sitting;

d alcohol intake, derived from self-reported dietary intake;

e self-reported at baseline;

f nSES = neighborhood socioeconomic status, derived fom cencus block group data, using indicators of education, occupation, unemployment, household income, poverty, and rental and house values;

g joint SES exposure derived from nSES Quintiles and Education variables as listed in the table

**Table 1B.** Characteristics of participants in Hawaii, by race and ethnicity, MEC (1993–1996)

	Hawaii							
	Race and Ethnicity, N (%)							
	Japanese American		Native Hawaiian		White		Total	
**Characteristic**	38786	(47.7)	12170	(15.0)	30427	(25.9)	81383	(100.0)
Age (years)								
mean (sd)	60.4	(9.1)	56.0	(8.5)	57.7	(9.1)	58.8	(9.2)
Sex, N (%)								
male	18438	(47.5)	5364	(44.1)	15509	(51.0)	39311	(48.3)
female	20348	(52.5)	6806	(55.9)	14918	(49.0)	42072	(51.7)
Marital Status, N (%)								
married	30167	(77.8)	8516	(70.0)	21004	(69.0)	59687	(73.3)
single	2639	(6.8)	722	(5.9)	2033	(6.7)	5394	(6.6)
separated, divorced, or widowed	5980	(15.4)	2932	(24.1)	7390	(24.3)	16302	(20.0)
BMI^a^ (kg/m^2^)								
mean (sd)	24.5	(3.8)	29.0	(6.2)	26.0	(4.8)	25.7	(4.9)
Diet Quality (HEI-2015 units^b^)								
mean (sd)	66.9	(10.7)	65.5	(11.0)	69.4	(10.5)	67.6	(10.7)
Physical Activity (METS/day^c^)								
mean (sd)	1.6	(0.3)	1.6	(0.3)	1.6	(0.3)	1.6	(0.3)
Alcohol intake (g/day^d^)								
mean (sd)	6.4	(19.2)	9.1	(27.2)	15.5	(29.2)	10.2	(25.0)
Smoking Status, N (%)								
Non-smoking	34147	(88.0)	9436	(77.5)	25456	(83.7)	69039	(84.9)
never smoked	19519	(50.3)	4758	(39.1)	11590	(38.1)	35867	(44.1)
formerly smoked	14628	(37.7)	4678	(38.4)	13866	(45.6)	33172	(40.8)
Current smoking	4639	(12.0)	2734	(22.5)	4971	(16.0)	12344	(15.1)
Cigarettes per day, among individuals who reported they smoke								
mean (sd)	16.5	(7.6)	16.6	(7.8)	19.3	(8.1)	17.6	(7.9)
Work Status (employment in a manufacturing enterprise and occupational category), N (%)								
No and office	25048	(64.6)	6416	(52.7)	21125	(69.4)	52589	(64.6)
No and labor/craft	2458	(6.3)	1287	(10.6)	1159	(3.8)	4904	(6.0)
No and office/labor/craft	5714	(14.7)	2898	(23.8)	4576	(15.0)	13188	(16.2)
Yes and office	949	(2.4)	311	(2.6)	505	(1.7)	1765	(2.2)
Yes and labor/craft	2290	(5.9)	557	(4.6)	1997	(6.6)	4844	(6.0)
Yes and office/labor/craft	2327	(6.0)	701	(5.8)	1065	(3.5)	4093	(5.0)
Education, N (%)								
Low Education (≤ high school)	15906	(41.0)	6311	(51.9)	6623	(21.8)	28840	(35.4)
High Education (> high school)	22880	(59.0)	5859	(48.1)	23804	(78.2)	52543	(64.6)
nSES Quintile^e^, N (%)								
Low nSES (Quintiles 1–3)	15693	(40.5)	6524	(53.6)	12078	(39.7)	34295	(42.1)
High nSES (Quintiles 4–5)	23093	(59.5)	5646	(46.4)	18349	(60.3)	47088	(57.9)
Joint SES Exposure^f^, N (%)								
1. Low nSES / Low Education	7505	(19.3)	3762	(30.9)	3218	(10.6)	14485	(17.8)
2. Low nSES / High Education	8188	(21.1)	2762	(22.7)	8860	(29.1)	19810	(24.3)
3. High nSES / Low Education	8401	(21.7)	2549	(20.9)	3405	(11.2)	14355	(17.6)
4. High nSES / High Education	14692	(37.9)	3097	(25.4)	14944	(49.1)	32733	(40.2)

Notes:

a BMI: Body Mass Index, derived from self-reported weight in kg divided in self-reported height in meters squared;

b units = points on the healthy eating index-2015: 0–100; Krebs-Smith et al., 2018; Park et al., 2021;

c metabolic equivalents of a task (METS) for activities in a typical 24 hour day, relative to 1 for sitting;

d alcohol intake, derived from self-reported dietary intake;

e self-reported at baseline;

f nSES = neighborhood socioeconomic status, derived fom cencus block group data, using indicators of education, occupation, unemployment, household income, poverty, and rental and house values;

g joint SES exposure derived from nSES Quintiles and Education variables as listed in the table

**Table 2.** Joint association of education and nSES with smoking prevalence at baseline, by location, MEC (1993–1996)

	California					
	Non-smoker (N)	Smoker (N)	Minimal Model^a^		Adjusted Model^b^	
			PR (95%CI)	*p*-value^d^	PR (95%CI)	*p*-value^d^
						
High nSES / High Education	16798	2281	ref	ref	ref	ref
High nSES / Low Education	7344	1198	1.44 [1.35, 1.53]	< .0001	1.29 [1.21, 1.37]	< .0001
Low nSES / High Education	20311	4598	1.44 [1.37, 1.52]	< .0001	1.33 [1.27, 1.40]	< .0001
Low nSES / Low Education	26327	6235	1.83 [1.74, 1.93]	< .0001	1.50 [1.42, 1.58]	< .0001
						
Omnibus *p*-value^c^			< .0001		< .0001	
Interaction *p*-value^e^			0.0008		0.0007	
						
	Hawaii					
	Non-smoker (N)	Smoker (N)	Minimal Model^a^		Adjusted Model^b^	
			PR (95%CI)	*p*-value^d^	PR (95%CI)	*p*-value^d^
						
High nSES / High Education	28619	4114	ref	ref	ref	ref
High nSES / Low Education	12027	2328	1.60 [1.52, 1.67]	< .0001	1.36 [1.30, 1.43]	< .0001
Low nSES / High Education	16581	3229	1.26 [1.19, 1.34]	< .0001	1.20 [1.14, 1.27]	< .0001
Low nSES / Low Education	11812	2673	1.73 [1.63, 1.83]	< .0001	1.41 [1.33, 1.49]	< .0001
						
Omnibus *p*-value^c^			< .0001		< .0001	
Interaction *p*-value^e^			< .0001		< .0001	

*Notes:* Non-smoker = individuals who used to smoke and those who never smoked; Smoker = individuals self-reported as having smoked > 20 packs of cigarettes in their lifetime and currently still smoke; nSES = neighborhood socioeconomic status, derived from census block group data, using indicators of education, occupation, unemployment, household income, poverty, and rental and house values; joint SES exposure derived from nSES Quintiles (1–3 Low and 4–5 High) and Education variables (Low Education: ≤ high school/12 years; High Education: > high school/12 years);

a The model is adjusted for Age at Cohort Entry, Sex, Race and Ethnicity, and clustering by census block group;

b The model is adjusted for Age at Cohort Entry, Sex, Race and Ethnicity, Marital Status, Physical Activity, Occupation, Alcohol intake, Body Mass Index, Diet Quality, and clustering by census block group.

c Omnibus Wald test (df = 3) to evaluate significance with smoking across levels of the joint SES exposure variable;

d Wald test (df = 1) to evaluate significance with smoking for individual levels of joint SES exposure vs. referent;

e Wald test for interaction (df = 1) comparing the joint effects model (having both exposures together) with the corresponding main effects model (having the linear combination of these exposures, or both exposures independently).

## Availability of data and material:

The dataset supporting the conclusions of this article is available by request from the authors, or by request through the following website: https://www.uhcancercenter.org/for-researchers/mec-data-sharing. All approved projects are listed with the name of the lead investigator for the project. The dataset used for this analysis is not permitted to be used for any other purpose without prior written approval from the MEC Research Committee.

## Abbreviations

BMI: body mass index
CA: California
CI: confidence interval
CPD: cigarettes per day
HEI: healthy eating index
HI: Hawaii
LA: Los Angeles
MEC: Multiethnic Cohort
MET: metabolic equivalents of a task
nSES: neighborhood socioeconomic status
PR: prevalence ratio
SES: socioeconomic status
SCCS: Southern Community Cohort Study
US: United States
