# Supplementary material for: Joint Association of Education and Neighborhood Socioeconomic Status with Smoking Behavior: The Multiethnic Cohort Study
Source: Res Sq. 2024 Nov 1:rs.3.rs-5281444. Preprint. [Version 1] doi: 10.21203/rs.3.rs-5281444/v1 (PMC11581112; doi:10.21203/rs.3.rs-5281444/v1)
Supplement: Supplement 1 [file NIHPPRS5281444V1-supplement-1.pdf]

Supplementary Files

This is a list of supplementary files associated with this preprint. Click to download.

- SupplementalTables10.8.24.xlsx
